# Glucagon-Like Peptide-1 and Diabetes 2012

**DOI:** 10.1155/2012/768760

**Published:** 2012-12-31

**Authors:** Matteo Monami, Giovanni Di Pasquale, Anna Rowzee, Carlo Maria Rotella, Edoardo Mannucci

**Affiliations:** ^1^Section of Geriatric Cardiology and Medicine, Careggi Teaching Hospital, Via delle Oblate 4, 50141 Florence, Italy; ^2^National Institute of Dental and Craniofacial Research, National Institutes of Health, Bethesda, MD, USA; ^3^Obesity Agency, Careggi Teaching Hospital, Florence, Italy; ^4^Diabetes Agency, Careggi Teaching Hospital, Florence, Italy

The present special issue was aimed at elucidating the role and the effects of glucagon-like peptide-1 (GLP-1) in type 2 diabetes. GLP-1 is a gastrointestinal hormone, mainly secreted after meals, capable of increasing glucose-stimulated insulin release and inhibits food intake. The physiological activity of this hormone has been demonstrated to be impaired in obese subjects and in patients with type 2 diabetes in comparison with healthy subjects.

Available data suggest that GLP-1 plays a relevant role in the regulation of postprandial glucose metabolism in physiologic conditions. Several new drugs act through the GLP-1 signaling system to stimulate insulin release and regulate blood glucose levels in patients with diabetes. Therefore, a special issue exploring the physiological properties of GLP-1 and the possible applications in several clinical settings is particularly warranted. 

This special issue is composed of 5 articles: two mechanistic studies and three systematic reviews and meta-analyses, exploring GLP-1-induced signaling mechanisms and molecular identification and cloning of the GLP-1 receptor. The reviews and meta-analyses are focused on the promising beneficial extraglycaemic effects of the incretin-based therapy, including those on lipid profile and cardiovascular risk.

*Matteo Monami* *Matteo Monami* *Giovanni Di Pasquale* *Giovanni Di Pasquale* *Anna Rowzee* *Anna Rowzee* *Carlo Maria Rotella* *Carlo Maria Rotella* *Edoardo Mannucci* *Edoardo Mannucci*

